# Supplementary material for: Horizontal antimicrobial resistance transfer drives epidemics of multiple Shigella species
Source: Nat Commun. 2018 Apr 13;9:1462. doi: 10.1038/s41467-018-03949-8 (PMC5899146; doi:10.1038/s41467-018-03949-8)
Supplement: Supplementary file 1 — Supplementary Information [file 41467_2018_3949_MOESM1_ESM.pdf]

Horizontal antimicrobial resistance transfer drives epidemics of multiple Shigella species

Baker et al.

Supplementary information

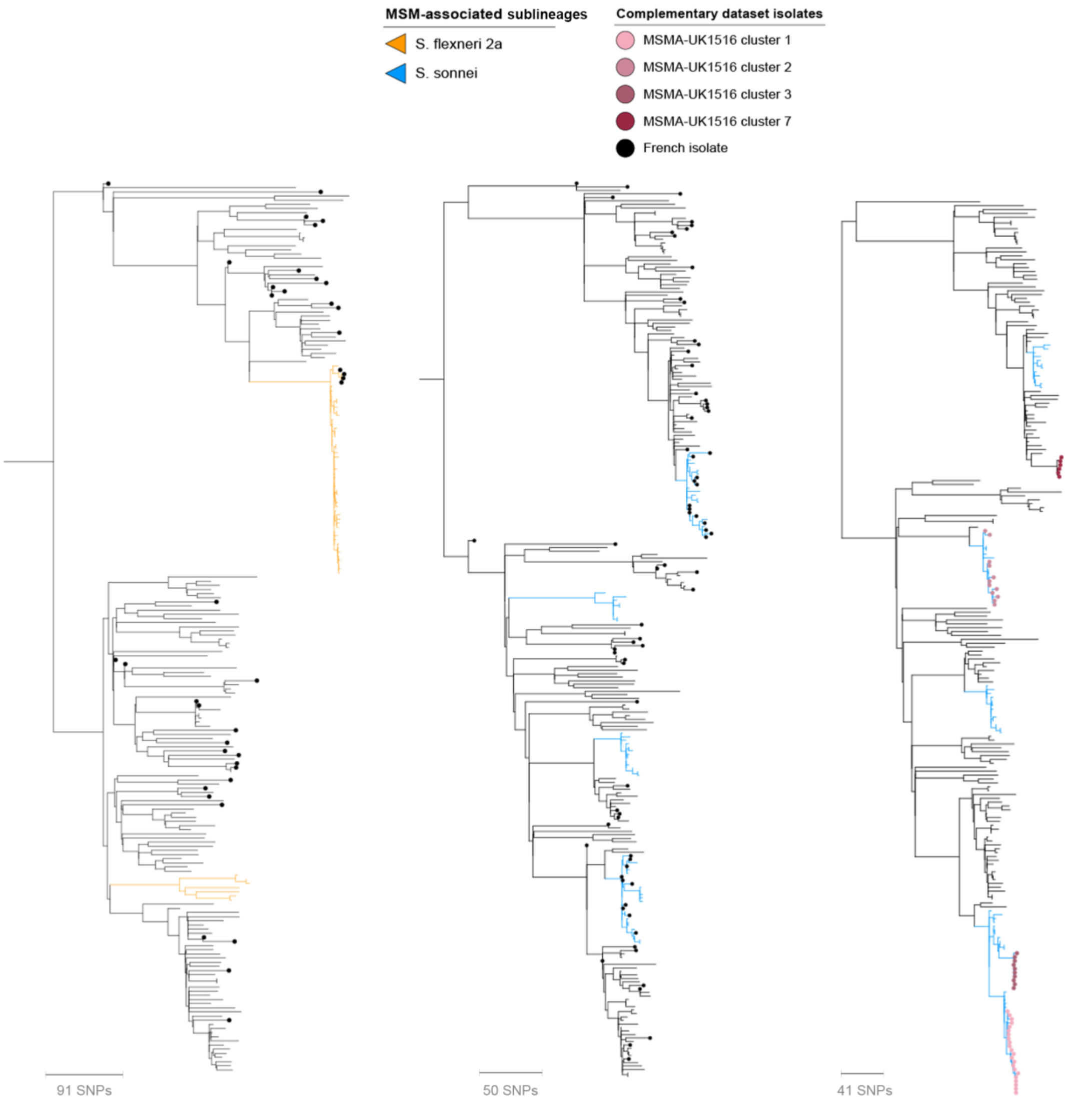

Supplementary Figure 1. Relationship of complementary isolates to MSMA sublineages. French cross sectional isolates of *S. flexneri* 2a and *S. sonnei* (left) and MSMA clusters of *S. sonnei* identified in the UK in 2015/16 (MSMA-UK1516, right) in relation to 2008 – 2014 representative subsample isolates. The whole genome phylogenies are mid-point rooted maximum-likelihood phylogenetic trees with scale bars in Single Nucleotide Polymorphisms (SNPs) are shown below each phylogeny. They show the position of complementary dataset isolates and MSMA sublineages according to the inlaid key. The rightmost tree is that used in Figure 3 of the main text.

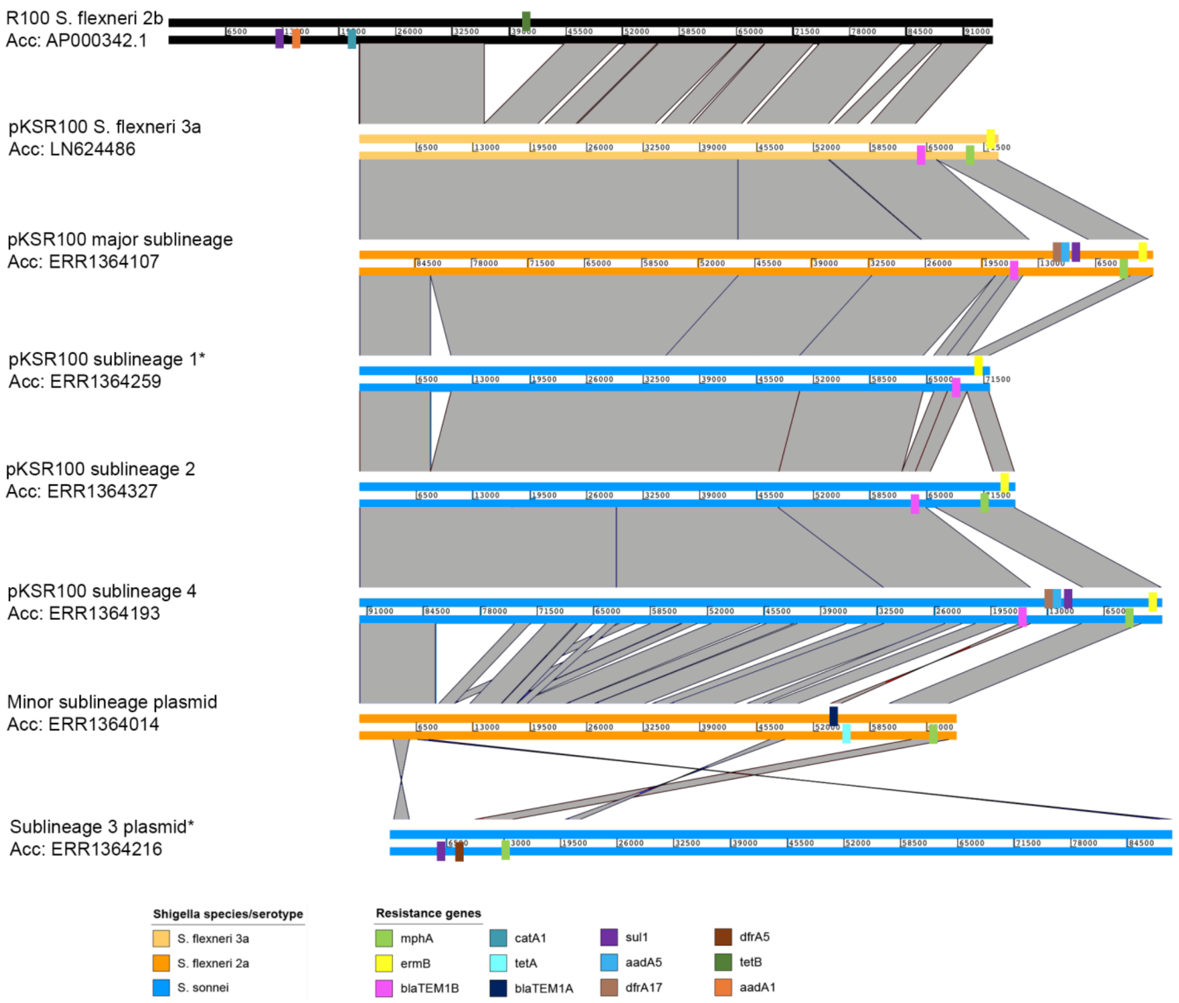

Supplementary Figure 2. Comparison of azithromycin resistance plasmid sequences from MSMA sublineages and R100. Linearised plasmid sequences are shown as labelled with intervening blocks showing regions of genomic synteny. The locations of AMR genes are shown according to the inlaid key. Those sequences that were generated by *de novo* assembly of short-read sequencing data (compared with SM-RT sequencing) are indicated by an asterisk.

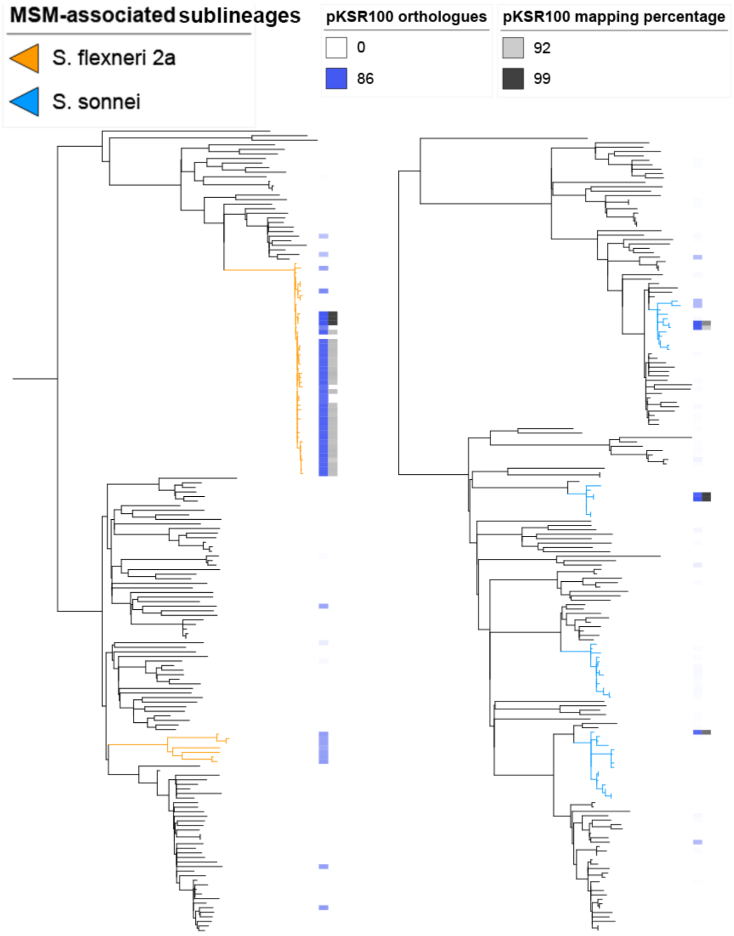

Supplementary Figure 3. Detection of pKSR100 among English representative subsample isolates. The phylogenetic trees (from Figure 1) are shown with adjacent tracks showing heat maps of the percentage of orthologues and mapping coverage of isolates containing ≥60 orthologues of pKSR100.

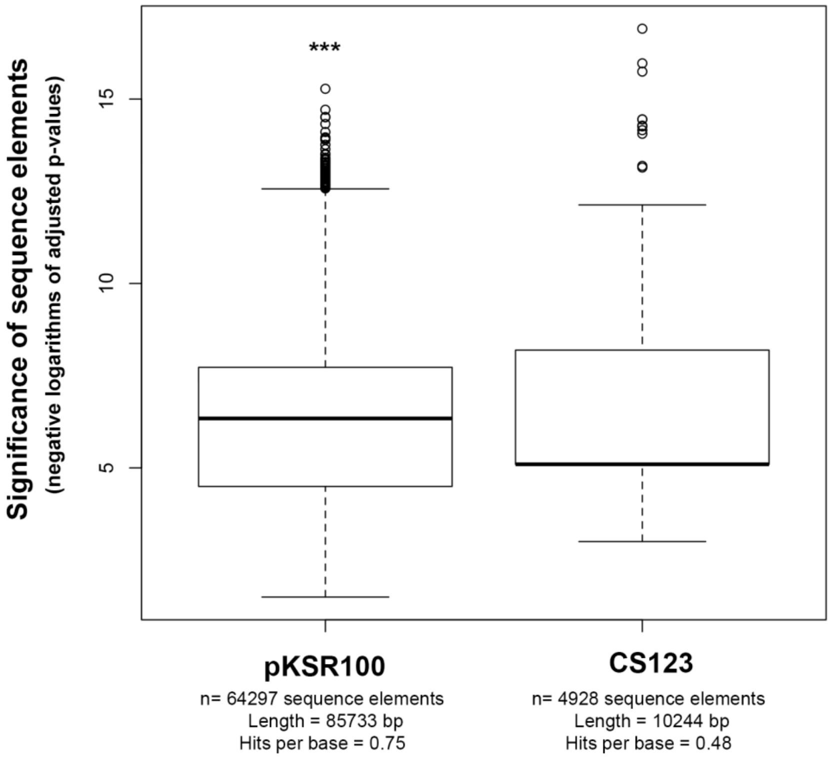

Supplementary Figure 4. Comparison of sequence element significances between pKSR100 and contiguous sequence 123 (CS123). The box plot shows the distribution of significance values of the sequence elements for each contiguous sequence, For each distribution, the central line represents the median, with the box hinges representing the first and third quartiles. The extent of values lying within 1.5 times the interquartile range are shown by the whiskers, and values beyond this (outliers) are shown by hollow circles. The significance difference of means between these two distributions (p-value <2.2 e-16, Welch Two Sample t-test) is indicated by the asterisks above the pKSR100 boxplot. The numbers of mapping sequence elements, length of the contiguous sequences, and calculated hits per base are shown below.

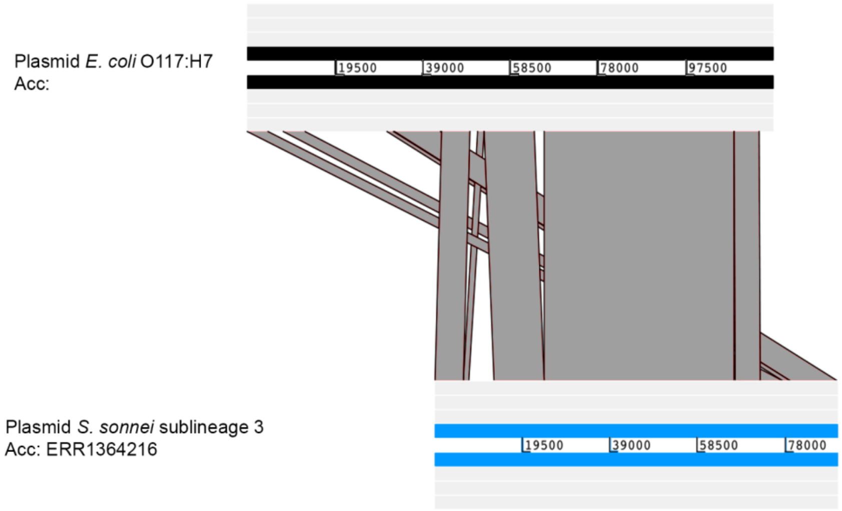

Supplementary Figure 5. Comparison of *E. coli* and *S. sonnei* plasmid sequences from MSMA outbreaks. The linearised plasmid sequences are shown according to the inlaid key with intervening blocks indicating genome synteny.

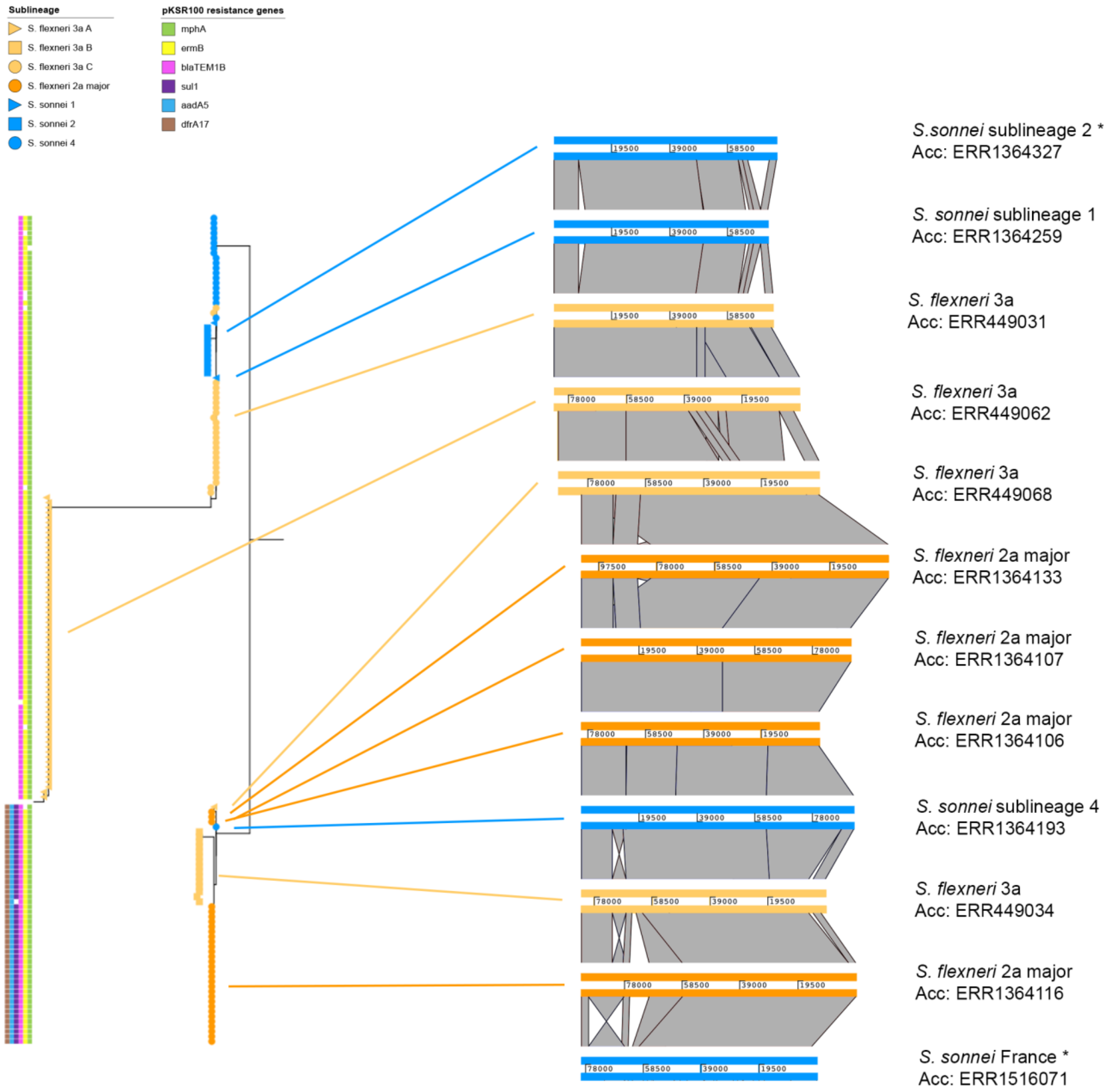

Supplementary Figure 6. pKSR100 diversity among *Shigella* sp.

The phylogenetic tree (left) is an enlargement of the pKSR100 phylogeny shown in Figure 3, with the genome sublineage represented at the tree tips and the presence of resistance genes found in pKSR100 shown in the leftmost tracks, both according to the inlaid keys. To the right is a comparison (as above) of circularised, full-length (SMRT-sequenced) pKSR100 sequences from various isolates throughout the tree (except where asterisked, which are contiguous sequences generated from *de novo* assembled Illumina short-read sequence data, see Methods). The phylogenetic positions of individual pKSR100 sequences are indicated by lines between the sequence and tree tip.

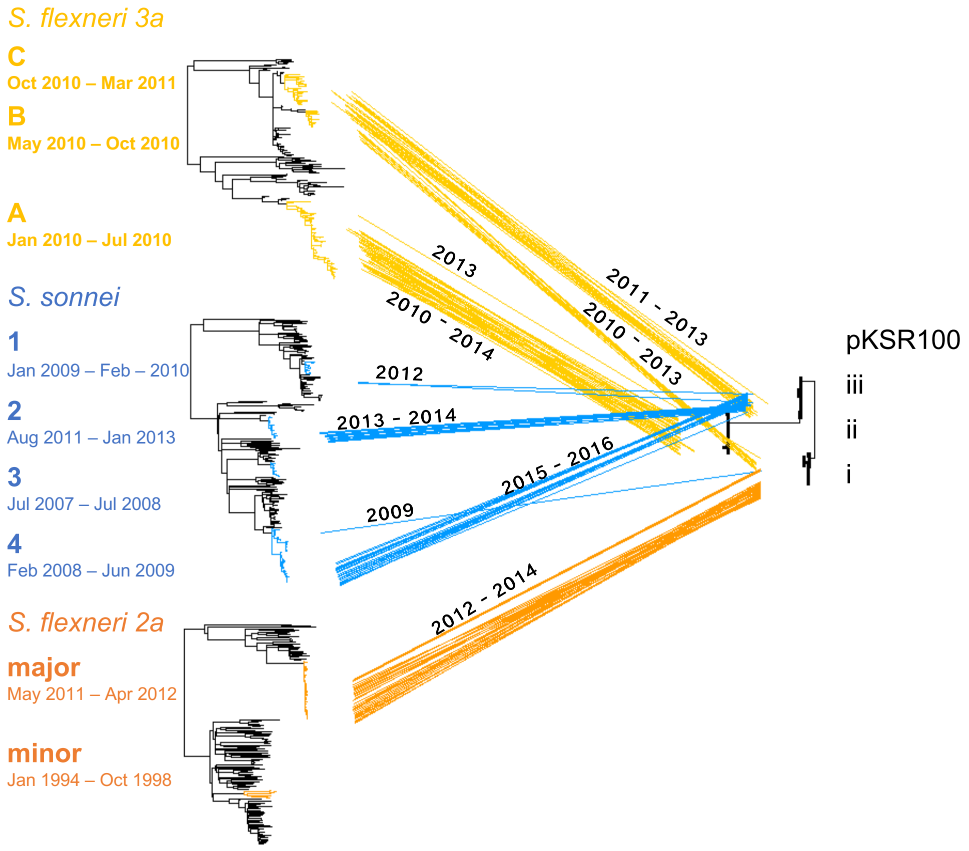

Year (HGT count)

2009 - 2010: *S. sonnei* sublineage 4 transfers pKSR100 variant i to *S. flexneri 3a* sublineage B **(1)**

2010 - 2011: In the already-epidemic (initially driven by behavioral and other factors) *S. flexneri 3a*, pKSR100 horizontally transmits twice **(2, 3)** to *S. flexneri 3a* sublineages A and C, and diversifies to form variants ii and iii. Epidemic is enhanced by pKSR100

2012: *S. flexneri 3a* sublineage B donates pKSR100 variant i to *S. flexneri* 2a, facilitating epidemic **(4)**. *S. flexneri 3a* sublineage C donates pKSR100 variant iii to *S. sonnei* clade 1 **(5)**.

2013: *S. sonnei* sublineage 2 receives pKSR100 variant iii from *S. flexneri* 3a sublineage C or *S. sonnei* sublineage 1 **(6)**. *S. flexneri 3a* sublineage A receives pKSR100 variant i from *S. flexneri 2a* or from *S. flexneri* 3a sublineage B **(7)**

2015: *S. sonnei* sublineage 4 (clusters 1 and 3 in 2015/16 UK MSMA isolates) receive pKSR100 variant iii from either *S. sonnei* sublineage 2 or *S. flexneri 3a* sublineage C (though in 2015, the former was not detected and the latter was not sampled for) **(8)**

Supplementary Figure 7. Combined genomic-epidemiological model of the impact of HGT of pKSR100 among shigellae. This is an elaboration of Figure 3 of the main text. The MSMA sublineages are labelled under the *Shigella* species/serotype headings with the 95% HPDs of the MRCAs shown in Figure 2 below, and the pKSR100 variants have been named to facilitate the below explanation. Underneath is a static explanation of the supplementary animation (Supplementary Movie 1) indicating the year and an increasing counter of the number of HGT events.

Species	Group	Year	2008	2009	2010	2011	2012	2013	2014
S. sonnei	**Cases**	**Travel**	287	337	369	307	227	281	266
		**Non-travel**	295	318	427	350	302	376	530
	**Sequenced isolates**	**Travel**	6	6	11	6	6	4	6
		**Non-travel**	18	22	23	22	18	13	26
***S. flexneri* 2a**	**Cases**	**Travel**	66	51	55	59	44	42	51
		**Non-travel**	82	84	103	93	85	118	255
	**Sequenced Isolates^**	**Travel**	6	6	11	6	6	4	6
		**Non-travel**	18	22	23	22	18	13	26

^ because of the greater number of total cases of *S. sonnei* and the epidemic transmission in adults, sequencing was restricted to isolates from patients aged 16 – 60 years old

Supplementary Table 1. Number of *Shigella* cases and isolates sequenced per years stratified by travel.

	Total	Largest pairwise dist.	Male patients	Low risk travel history	16 – 60 years old
***S. flexneri* 2a**					
All (PG3)	176	552	104	146	66
Major sublineage	47	20	45 **	46 *	45 **
Minor sublineage	7	148	7 *	7 *	7 *
S. sonnei					
All (Lin. III)	181	395	97	138	All
Sublineage 1	12	25	11 *	10	-
Sublineage 2 ^#	8	15	7	8	-
Sublineage 3	13	21	13 *	13	-
Sublineage 4 ^	16	30	16 *	16 *	-

Supplementary Table 2. Patient demographic compositions and associations of phylogenies and MSMA sublineages. Asterisks show significance of sublineage association with demographic characteristic (**p< 0.0001, *p<0.05, Fisher's exact test). Some clusters (^) were phylogenetically equivalent to MSMA clusters circulating in the UK in 2016 and *S. sonnei* sublineage 2 (#) was designated as an MSM-associated sublineage on this basis

NB: Another *S. flexneri* 2a sublineage was investigated for statistical significance of over-representation of the above demographic characteristics. This was comprised of five isolates from 16 – 60 years old male patients with no history of recent travel and is positioned between the major and minor sublineage on the phylogenetic tree. However, this failed to reach statistical significance as above so was not designated as MSMA.

**MSMA sublineage**	**Isolate(s)**	**MIC (mg/L)**
Intercontinental *S. flexneri* 3a	*mphA*and *ermB* (n=4)	64 – >256
	mphA only (n=1)	16
S. flexneri 2a major sublineage	No macrolide resistance genes (n=5)	<0.5 - 1
	mphA and *ermB* (n=11)	64 - >128
S. flexneri 2a minor sublineage	No macrolide resistance genes (n=1)	<0.5
	mphA only (n=5)	16 - 32
S. sonnei sublineage 1	No macrolide resistance genes (n=4)	2
S. sonnei sublineage 2	No macrolide resistance genes (n=3)	1 - 2
	mphA and *ermB* (n=1)	>128/128
S. sonnei sublineage 3	No macrolide resistance genes (n=2)	1 - 2
	mphA (n=4) *	2 - 8
S. sonnei sublineage 4	No macrolide resistance genes (n=4)	1 - 2
	mphA and *ermB* (n=1)	>256
S. flexneri 2a, non MSMA sublineages	No macrolide resistance genes (n=12)	<0.5 - 1
	mphA (n=3)	<0.5 - 16
S. sonnei, non-MSMA sublineages	No macrolide resistance genes (n=12)	0.5 - 2
	mphA and *qepA* (n=1)	32

* These isolates had lower-than anticipated MICs given that the *mphA* gene was found among their assembled short-read sequencing data. However, ERR1364216 was among this subset and underwent SMRT sequencing after which assembly analysis showed the plasmid (and *mphA* gene) was absent. This indicates the possible loss of this plasmid by *S. sonnei* on repeat culture.

Supplementary Table 3. Azithromycin phenotype testing of isolates in this study.

Patient	Isolate 1	Isolate 2	Intervening time	Genetic distance
1	*S. flexneri* 3a<br>MSMA, no sublineage	*S. flexneri* 3a<br>MSMA, no sublineage	9 days	3 SNPs
2	*S. flexneri* 3a<br>MSMA sublineage A	*S. flexneri* 3a<br>MSMA sublineage A	20 days	4 SNPs
3	*S. flexneri* 3a<br>MSMA sublineage A	*S. flexneri* 3a<br>MSMA sublineage A	36 days	0 SNPs
4	*S. flexneri* 3a<br>MSMA sublineage A	*S. flexneri* 3a<br>MSMA sublineage A	49 days	0 SNPs
5	*S. flexneri* 3a<br>MSMA sublineage A	*S. flexneri* 3a<br>MSMA sublineage A	95 days	2 SNPs
6	*S. flexneri* 3a<br>MSMA, no sublineage	*S. flexneri* 3a<br>MSMA, no sublineage	154 days	118 SNPs
7	*S. sonnei*<br>MSMA sublineage 4	*S. flexneri* 2a major<br>MSMA sublineage	318 days	Different species
8	*S. flexneri* 3a<br>MSMA sublineage	*S. sonnei*<br>non-MSMA	430 days	Different species
9	*S. flexneri* 3a<br>MSMA, no sublineage	*S. flexneri* 3a<br>MSMA sublineage C	524 days	18 SNPs
10	*S. flexneri* 3a<br>MSMA sublineage A	*S. flexneri* 3a<br>MSMA, no sublineage	911 days	128 SNPs

Supplementary Table 4. Epidemiological details of patients from whom *Shigella* were serially isolated. All patients were 16 – 60 years old males without a recent history of travel to regions endemic for shigellosis. NB: for patients 1 – 5 it is possible that these represent chronic, rather than repeat infections as they were closely related (0-4 SNPs) phylogenetically.
