# Supplementary material for: Horizontal antimicrobial resistance transfer drives epidemics of multiple Shigella species
Source: Nat Commun. 2018 Apr 13;9:1462. doi: 10.1038/s41467-018-03949-8 (PMC5899146; doi:10.1038/s41467-018-03949-8)
Supplement: Supplementary file 3 — Description of Additional Supplementary Files [file 41467_2018_3949_MOESM3_ESM.pdf]

Description of Additional Supplementary Files

File Name: Supplementary Movie 1

Description: Animation of horizontal transmission of pKSR100.

File Name: Supplementary Data 1

Description: Metadata, accessions, and results for isolates used in this study.
